# Considerations in using heart rate-based physical activity estimates from consumer wearables in individuals with varying weight status

**DOI:** 10.1186/s12966-025-01801-z

**Published:** 2025-07-28

**Authors:** Denver M. Y. Brown, David Wing, Christopher D. Pfledderer, Peter Stoepker, Stuart J. Fairclough, Jordan A. Carlson

**Affiliations:** 1 Department of Kinesiology, Kansas State University, Manhattan, KS USA; 2 Herbert Wertheim School of Public Health and Human Longevity Science, University of California San Diego, La Jolla, CA USA; 3 School of Public Health in Austin, The University of Texas Health Science Center Houston, Austin, TX USA; 4 Sport, Physical Activity, Health, and Wellbeing Research Group, Department of Sport and Physical Activity, Edge Hill University, Ormskirk, UK; 5 International Centre for Applied Research with Children, Young People, Pregnant Women and Families (iCARE), Edge Hill University, Ormskirk, UK; 6 Center for Children’s Healthy Lifestyles & Nutrition, Children’s Mercy Hospital, Kansas City, MO USA; 7 Exercise and Physical Activity Resource Center, University of California San Diego, La Jolla, CA USA

**Keywords:** Wearable devices, Fitness, Adiposity, Relative intensity, Absolute intensity, Accelerometry

## Abstract

Although moderate-to-vigorous physical activity (MVPA) is a widely used construct in physical activity (PA) research, the lack of standardized assessment methods– particularly with the growing use of consumer-grade wearable activity trackers– poses challenges for comparability. Consumer-grade devices tend to rely on heart rate (HR)-based estimation methods to classify PA intensity, which contrasts with traditional research-grade accelerometers that use count- or raw-acceleration metrics. Comparability issues are particularly salient across individuals with varying weight status. In this commentary, we discuss systematic discrepancies between HR-based (relative intensity) and acceleration-based (absolute intensity) classifications of MVPA among individuals with differing weight statuses. Using Fitbit data from the Adolescent Brain Cognitive Development Study, we illustrate how HR-based PA intensity classification may indicate higher MVPA in youth with greater adiposity despite lower step counts and light PA levels. We highlight implications for research design, public health surveillance, messaging, policy, and interventions. We also call for greater transparency, standardized methodologies, and integrative measurement approaches to ensure more accurate assessment of PA behavior.

Moderate-to-vigorous physical activity (MVPA) is a widely used construct in physical activity (PA) research, yet there is no single standardized method for its assessment with variability based on several factors such as device type, placement, and data processing decisions. Consequently, truly valid comparisons are only possible when MVPA is assessed, defined, and reported consistently. With the rise of consumer-grade wearable activity trackers, considerations over comparability have become even more relevant but also more challenging.

Consumer wearables (e.g., Fitbit, Apple Watch, Garmin) are increasingly deployed to study PA behavior(s). These devices offer some advantages over research-grade accelerometers, including the ability to support behavior change interventions, continuous wear that enhances data reliability and allows for long-term behavioral insights, and being cost-effective in large-scale studies. However, a key distinction between consumer wearables and most research-grade accelerometers is their approach to PA intensity estimation– whether through absolute or relative intensity-based methods. These differences must be carefully considered when using consumer devices for research.

While most research-grade accelerometers classify PA using count- or raw acceleration-based absolute intensity methods [1], consumer wearables with heart rate (HR) sensors typically derive intensity from HR [2]. This distinction introduces two primary ways of assessing PA intensity: (1) Absolute intensity, determined by movement (e.g., acceleration-based), anchored to an empirically-derived intensity threshold (e.g., 200 m*g* average acceleration), and applied to all study participants; and (2) Relative intensity, which reflects individual physiological response to movement (e.g., HR-based). Theoretically, the relative intensity method adjusts for individual differences in cardiorespiratory fitness and physiological adaptation. International (i.e., World Health Organization) and national public health recommendations for PA (e.g., PA Guidelines for Americans, 2nd ed) generally describe MVPA in terms of both absolute and relative intensity, though more attention has been given to absolute intensity by drawing on population-average metabolic equivalent of task (MET) values for common activities (e.g., walking at 3.0 miles per hour requires 3.5 METs and thus meets the 3.0 METs threshold for MVPA). This has helped contribute to clear and understandable PA guidelines at the population level. When considering relative intensity, however, a given volume of movement may reach the MVPA threshold for one individual but not another. Relative intensity is most commonly derived based on age-predicted HR or HR reserve, which is defined based upon a percentage of the difference between maximal (usually estimated) HR and resting HR. Typically, using this method, moderate intensity is considered to begin at 40% of HR reserve [3], which will result in variable absolute intensity based on a variety of physiologic factors.

Thus, while both absolute- and relative-intensity methods assume a linear relationship between movement and intensity, HR responses to PA are not always consistent across individuals with differing physiological characteristics (e.g., fitness level, adiposity). This has been shown to lead to systematic differences in estimation of MVPA when using relative compared to absolute measurement tools [4]. These measurement differences are particularly relevant given the growing global prevalence of obesity across all age groups [5], where PA classification methods could influence how PA levels are estimated across weight statuses. Individuals with obesity exhibit distinct physiological characteristics– including higher resting HR, lower cardiovascular efficiency, and altered metabolic responses to PA that tend to result in higher HR at lower movement intensities. This can result in individuals with obesity who have lower fitness reaching the HR-based (relative) threshold for MVPA without reaching the movement-based (absolute) threshold.

Evidence of this issue is demonstrated by an analysis of the Year 2 Follow-Up wave of the Adolescent Brain Cognitive Development (ABCD) Study (*n* = 5793) involving MVPA data based on Fitbit’s HR-based scoring algorithm. Adolescents classified as obese (M = 46 ± 36 SD min/day) or overweight (M = 37 ± 30 SD min/day) appeared to accumulate more MVPA than their healthy-weight peers (M = 31 ± 27 SD min/day), contradicting prior research indicating that youth with obesity typically engage in less MVPA [6]. This trend persisted when using an alternative adiposity indicator, abdominal obesity status (height/waist circumference > 0.5), with youth meeting this criterion averaging 42 ± 36 SD min/day of MVPA compared to 34 ± 31 SD min/day among their non-abdominally obese peers. Closer inspection of light PA (LPA) and step count data provided additional insights. Adolescents with excess adiposity engaged in less LPA (Obese: M = 258 ± 58 SD min/day; Overweight: M = 269 ± 54 SD min/day; Healthy weight: M = 283 ± 61 SD min/day) and took fewer daily steps (Obese: M = 8,603 ± 3,054 SD steps/day; Overweight: M = 8,942 ± 2,954 SD steps/day; Healthy weight: M = 9,445 ± 3,193 SD steps/day). These findings illustrate a dose-response relationship: as excess adiposity increases, estimates of LPA and step counts decrease, while MVPA estimates increase. This paradoxical pattern suggests that systematic differences in HR-based intensity classification may contribute to these discrepancies. Similar discrepancies have also been reported in adult studies using consumer wearables [4]. Related patterns have emerged in studies with research-grade HR sensors [7] and accelerometry [8, 9], where adults with higher BMI or lower cardiorespiratory fitness accumulated more MVPA than those with lower BMI or higher fitness when using relative-intensity classification, while the opposite was observed with absolute-intensity methods. Together, these findings underscore how the method of intensity classification can substantially alter observed relationships with fitness or adiposity, reinforcing the need to critically examine and reconcile these divergent approaches.

This need is especially apparent when considering the challenge of aligning HR- and movement-based PA intensity classifications derived from wearables, particularly in population groups with lower fitness. Notably, combining HR with accelerometry from research-grade devices has been shown to improve PA estimation accuracy [10], suggesting that similar approaches could enhance the validity of PA estimates from consumer wearables. Despite this potential, most studies continue to rely on PA estimates derived entirely or primarily from either HR or acceleration. Without careful consideration of these methodological differences, studies risk making misleading conclusions about PA intensity and weight status. These classification differences have important implications for public health surveillance, messaging, policy, and interventions, as overestimating MVPA in at-risk populations could misinform strategies aimed at promoting PA engagement. These differences are particularly relevant for researchers examining obesity as an outcome or studying PA differences by weight status (i.e., between-participant designs). The unexpected ABCD Study finding that HR-based MVPA was positively associated with adiposity could potentially mislead research on obesity-related disparities if not carefully considered within this measurement context. Classification methods may also influence comparisons across different activity intensities. For example, as PA research increasingly embraces integrative 24-hour approaches, methods such as compositional isotemporal substitution models might suggest that replacing sedentary behavior with MVPA is associated with increased adiposity, contradicting existing evidence [11]. 

Meanwhile, within-participant comparisons in longitudinal studies introduce additional complexities. Individuals who lose weight or improve fitness may show a reduction in HR-based MVPA despite increased movement, while those who gain weight may exhibit the opposite pattern. These physiological adaptations, which vary across individuals, can complicate interpretations of changes in PA behavior over time. At the same time, relative intensity-based classification offers important advantages by accounting for physiological capacity and the true cardiovascular burden of movement. It may reduce misclassification that can occur with absolute-intensity methods, whereby time spent in MVPA is misclassified as LPA among individuals with lower fitness, such as those with certain illnesses (e.g., COPD), physical disabilities (e.g., mobility impairments), or intellectual and developmental disabilities (e.g., Down syndrome). Additionally, assessing the relative intensity of an individual’s PA at baseline may help personalize interventions by identifying their “spare physical capacity” [12]. For example, individuals engaging in PA at a low relative intensity may have more capacity to increase intensity in response to an intervention, whereas individuals already engaging at a high relative intensity may require tailored strategies to avoid discouragement and sustain (rather than increase) their current intensity. Thus, relative-intensity measures should be considered in addition to absolute-intensity measures when personalizing and evaluating interventions. These considerations underscore the need for appropriate PA classification methods when assessing longitudinal changes. Transparent reporting of PA assessment methods and awareness of discrepancies between HR- and acceleration-based estimates will be critical for ensuring accurate interpretation and advancing our understanding of PA-adiposity relationships.

## Conclusion

As consumer wearables become more prevalent in PA research, particularly in large-scale studies, it is essential to critically assess measurement approaches and implications for data interpretation. The increased use of HR-based classification introduces unique challenges, particularly in individuals with excess adiposity, where classification differences may distort PA estimates and their associations with health outcomes. Researchers must be cautious to interpret their data within the context of the measurement approach used and PA guidelines as well as prior literature. Greater transparency from manufacturers regarding data processing methods is also critical to strengthen the reliability and comparability of PA estimates. Future research should systematically evaluate these discrepancies between relative- and absolute-intensity classification and establish best practices for comparing findings across the different methods as well as for potentially integrating HR- and acceleration-based PA estimates. To advance this work, more studies that investigate both relative and acceleration-based PA intensity estimates within the same individuals are critically needed. Such data would enable more precise identification of both group- (e.g., weight status, cardiorespiratory fitness) and individual-level (e.g., resting HR, movement efficiency) sources of discrepancy between classification methods. Approaches that could improve PA estimates with consumer monitors include: (1) applying scoring algorithms to raw accelerometer data [1], (2) combining accelerometer and HR data [10], and (3) using step cadence [13]. While raw acceleration data from wearables may help bridge the gap between consumer wearables and research-grade devices [14], many consumer monitor manufacturers make it difficult– or even impossible– to obtain raw data. Overall, these considerations in PA measurement underscore the need for standardized frameworks and opportunities for PA measurement specialists to improve classification accuracy. By advancing methodological consistency and promoting transparent PA classification, researchers can enhance the utility of consumer wearables while ensuring accurate and meaningful insights into PA behavior.

## Data Availability

The data and analyses presented in this commentary were used for illustrative purposes only. The underlying data are available through the NIMH Data Archive by submitting a request at https://nda.nih.gov/abcd/request-access. Code used to generate the illustrative analyses is available upon reasonable request from the corresponding author (DB).
